# Does vascular involvement affect erectile dysfunction in patients with Behçet's disease?: a single-center cross-sectional study

**DOI:** 10.1590/1516-3180.2025.3295.12062026

**Published:** 2026-09-21

**Authors:** Bünyamin Polat, Abdulsamet Erden, Selman Ünal, Hakan Apaydin, Berkan Armağan, Serdar Can Güven, Emrah Okulu, Ahmet Omma, Orhan Kucuksahin, Önder Kayigil

**Affiliations:** I Ankara Bilkent City Hospital, Department of Rheumatology, Ankara, Türkiye.; II Assoc. Prof.; Gazi University, Department of Internal Medicine, Division of Rheumatology, Ankara, Türkiye.; III Ankara Bilkent City Hospital, Urology Clinic, Ankara, Türkiye.; IV Assoc. Prof.; Ankara Etlik City Hospital, Department of Rheumatology, Ankara, Türkiye.; V Assoc. Prof.; Ankara Bilkent City Hospital, Department of Rheumatology, Ankara, Türkiye.; VI Assoc. Prof.; Ankara Bilkent City Hospital, Department of Rheumatology, Ankara, Türkiye.; VII Ankara Bilkent City Hospital, Urology Clinic, Ankara, Türkiye.; VIII Prof.; University of Health Sciences, Department of Rheumatology, Ankara, Türkiye.; IX Prof.; Yıldırım Beyazıt University, Department of Internal Medicine, Division of Rheumatology, Ankara, Türkiye.; X Prof.; Ankara Bilkent City Hospital, Urology Clinic, Ankara, Türkiye.

**Keywords:** Behçet's Disease, Erectile Dysfunction, Rheumatology, Sexual Dysfunction, Vasculitis, Hereditary Autoinflammatory Diseases, Male Impotence, Musculoskeletal Disorders

## Abstract

**BACKGROUND:**

Behçet's disease (BD) can affect the urogenital system in men, leading to conditions such as epididymitis, urethritis, cystitis, and dorsal penile vein thrombosis. A high incidence of erectile dysfunction (ED) in BD patients has been previously reported; however, the role of vascular involvement in ED is unclear.

**OBJECTIVES:**

To investigate the effect of vascular involvement on ED in male patients with BD.

**METHODS:**

Ninety-seven patients diagnosed with BD (mean age, 40.4 ± 10.7 years; disease duration, 12.5 ± 9.6 years) were included in the study and divided into the vascular and non-vascular groups. The presence and severity of ED were assessed using the International Index of Erectile Function-5 (IIEF-5), with a score of ≤21 indicating the presence of ED. Patients with ED underwent corpus cavernosum electromyography and penile color Doppler ultrasonography to diagnose ED.

**RESULTS:**

Half of all patients with BD (n = 49, 50.5%) were found to have ED. No significant difference in the presence of ED was observed between the two groups (vascular group: n = 24, 49% vs. non-vascular group: n = 25, 52.1%; p = 0.76). The proportion of patients with peak systolic velocities of less than 30 cm/s was significantly higher in the vascular group than in the non-vascular group (n = 12, 80% vs. n = 2, 22.2%; p = 0.01). No significant between-group difference was found in corpus cavernosum electromyography results (p > 0.05).

**CONCLUSIONS:**

ED is common in patients with BD, and arterial ED is more prevalent among those with vascular involvement. Sexual dysfunction, which is less frequently screened for in patients with BD, should be thoroughly monitored; early intervention is recommended.

## INTRODUCTION

Behçet's disease (BD) is a systemic vasculitis of unknown etiology that can affect blood vessels of any size and type.^1^ Its clinical features may include recurrent oral ulcers, genital ulcers, papulopustular lesions, uveitis, and thrombotic events.^2^ BD may also involve the neurological, gastrointestinal, ocular, articular, vascular, and urogenital systems.^3^ Cellular immunity is known to play a key role in the pathogenesis of BD, with macrophage activation, neutrophil chemotaxis, and phagocytosis being observed in BD lesions.^4^ Mucocutaneous lesions, such as oral ulcers, papulopustular lesions, and erythema nodosum, are considered to result from neutrophil activation, leading to tissue damage.^5^ Neutrophils have been found to dominate around the vasa vasorum in BD, releasing proinflammatory cytokines such as interleukin-1 alpha, tumor necrosis factor alpha, and interferon-gamma.^6^ Neutrophil activation and the formation of neutrophil extracellular traps are understood to play a crucial role in the vascular involvement observed in BD.^7^

The most common type of vascular involvement in BD is superficial and deep venous thrombosis, affecting approximately 15% to 40% of patients. The inferior and superior vena cava, portal vein, and cerebral sinuses can also be involved.^8^ Arterial involvement is less common, and usually limited to the peripheral, visceral, and pulmonary arteries.^9^ BD can also affect the urogenital system in male patients, causing conditions such as epididymitis, urethritis, cystitis, and dorsal penile vein thrombosis; these can lead to urogenital problems such as erectile dysfunction (ED).^10,11^ ED may result from damage caused by neurological and articular involvement in patients with BD, and also from the physical and psychological effects of these conditions. Furthermore, ED may be linked to the medications used for treatment and the inflammatory burden of the disease itself. Various factors such as age, education level, systemic diseases, and depression are also known to affect sexual dysfunction in patients with BD.^12^ Although several factors related to sexual dysfunction in BD have been identified, the literature regarding the effect of vascular involvement is limited.

Evidence indicates that sexual dysfunction is common in patients with BD,^13^ with a prevalence of sexual dysfunction in patients with BD of 68.7% being reported in one study.^14^ These findings were supported by those of another study, in which sexual dysfunction scores were reported to be higher in patients with BD than in the healthy population.^15^ Sexual dysfunction in BD may arise from organic factors, secondary psychiatric conditions, drug-related effects,^16^ or involvement of the central nervous system and urogenital organs.^17^ In BD, neurogenic impotence may occur with central nervous system involvement, whereas impotence due to vascular insufficiency is observed with vascular involvement.^17,18^ Venous insufficiency was also found in two cases of ED without neurological involvement.^16^ However, the authors noted that the role of venous insufficiency of the penis in ED is unclear. Therefore, in this study, we investigated the effect of vascular involvement on ED in male patients with BD.

## METHODS

### Study design

This single-center cross-sectional study was approved by the Institutional Review Board of Ankara Bilkent City Hospital (approval number E2-21-457). Written informed consent was obtained from all patients, and all procedures were conducted in accordance with the Declaration of Helsinki. This study was conducted at the Rheumatology and Urology Outpatient Clinics of Ankara Bilkent City Hospital between January and December 2022.

### Participants

Ninety-seven patients diagnosed with BD according to the criteria outlined in the International Criteria for Behçet's Disease^19^ were included in the study. Sexually active male patients with BD, aged 18 to 65 who were having sexual intercourse at least once a month were included in the study. Patients who refused urological evaluation; had a serious neurological (ischemic or thrombotic cerebrovascular event, multiple sclerosis, etc.), endocrinological (hypo- or hyperthyroidism, pituitary insufficiency, hypogonadism, etc.), vascular, or urological disorder unrelated to BD; experienced a hormonal disorder based on evaluations of luteinizing hormone, testosterone, estradiol, and prolactin; or had a history of malignancy were excluded from the study.

### Identification of the groups

**Figure 1:**
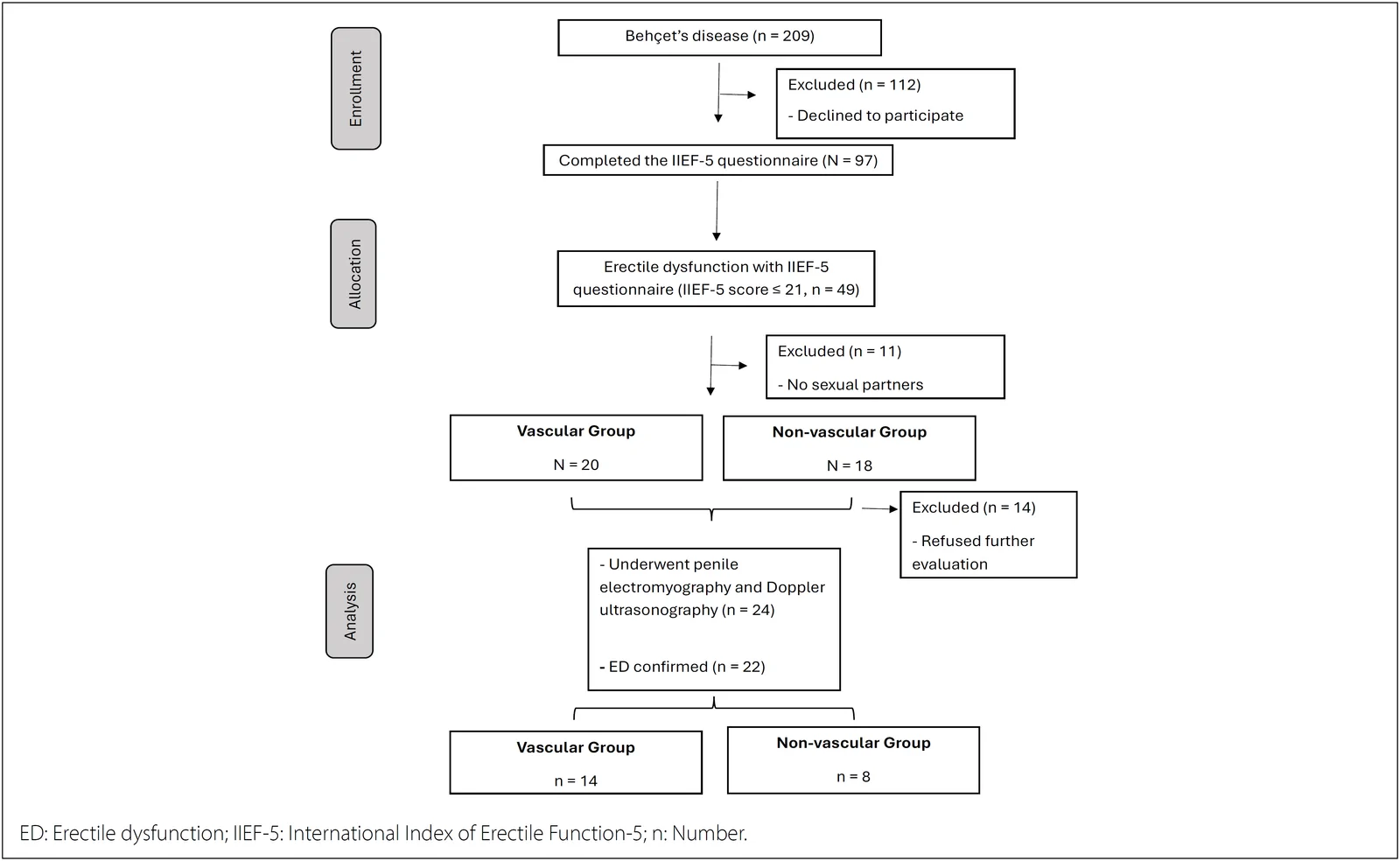
Flow chart of the study.

A total of 209 patients who met the inclusion criteria were informed about the study and invited to participate; of these, 97 agreed to participate. The patients with BD were divided into two groups: the vascular group (VG; n = 49) and non-vascular group (NVG; n = 48) (Figure 1). The presence of vascular involvement in patients with BD was determined using the hospital database. A history of deep vein thrombosis in the peripheral, central, or sinus veins; thrombosis or aneurysm in the pulmonary and extrapulmonary arteries (including the aorta, and peripheral and coronary arteries); and cardiac thrombus were classified as vascular involvement. Patients with such vascular involvement were included in the VG. Patients with no history or symptoms of vascular involvement or superficial thrombophlebitis were included in the NVG.

### Evaluations

Sociodemographic data, disease duration, comorbidities, smoking history, clinical features of the BD, HLA-B51, and active medical treatments for the BD were recorded. The presence of arthralgia, arthritis, oral ulcers, papulopustular lesions, erythema nodosum, uveitis, pathergy, thrombosis, aneurysms, and genital ulcers was evaluated. The level of depression was assessed using the Beck Depression Inventory.^20^ The total score on the Beck Depression Inventory ranges from 0 to 63, with a score of 17 or higher indicating the presence of moderate to severe depression.^20^ In our study, we accepted those with moderate and severe depression to be patients with depression.

### Erectile dysfunction

The presence and severity of ED were assessed using the International Index of Erectile Function-5 (IIEF-5).^21^ The IIEF-5 consists of five questions, each scored with between 1 and 5 points, with a total score calculated from the responses. An IIEF-5 score of 5 to 7 points indicates severe ED, 8 to 11 points indicates moderate ED, 12 to 16 points indicates mild to moderate ED, and 17 to 21 points indicates mild ED. Scores of 22 or above are not considered indicative of ED,^22^ thus patients with an IIEF-5 score of 21 or below were classified as having ED. The IIEF-5 score is an excellent diagnostic test, and a cutoff value of 21 points was found to have a specificity of 88% and sensitivity of 98%.^23^ The Turkish reliability study of the index was conducted by Turunç et al.^24^

After determining the status of ED, all patients with ED were invited to undergo corpus cavernosum electromyography (CC-EMG) and penile color Doppler ultrasonography (CDUS). However, only 24 patients chose to participate in these examinations: 11 patients had no sexual partner and 14 patients refused to participate. Penile CDUS and CC-EMG examinations were therefore performed on 24 patients with ED. These measurement results were compared based on vascular (n = 15) and non-vascular (n = 9) involvement.

### Corpus cavernosum electromyography

Electrical activity of the corpus cavernosum was measured using CC-EMG with a High Speed EMG Module (Medical Measurement Systems B.V., Enschede, Netherlands) connected to a computer system, as previously described.^25^ A coaxial needle electrode was placed on the flaccid penis, while a grounding electrode was placed on the patient's foot to prevent electrical interference. After recording the electrical activity for 10 minutes, measurements were continued after administering an intracavernosal injection of 60 mg of papaverine. All tests were carried out in a well-insulated room maintained at a temperature of between 20 and 24 °C. Patients were informed about the minimally invasive nature of the test and the absence of any electrical stimulation. The mean amplitude potential was calculated from peak to peak using the last 10 potentials. The amplitudes of the patients’ cavernous smooth muscle before and after injecting the papaverine were measured, and the degree of relaxation was calculated as follows: Degree of relaxation = (amplitude before papaverine injection − amplitude after papaverine injection) ÷ amplitude before papaverine injection).^2 6^

### Penile color Doppler ultrasonography

To determine the vascular etiology, blood flow in the cavernous arteries was measured using penile color Doppler ultrasonography (Voluson S8; GE Healthcare Technologies, Inc., Chicago). Following an intracavernosal injection of 60 mg of papaverine using a 27-gauge needle, blood flow measurements were repeated, and peak systolic and end-diastolic velocities were recorded. A peak systolic velocity greater than 30 cm/s and an end-diastolic velocity less than 3 cm/s were considered indicative of normal penile vascular flow.^27^ The resistive index was calculated using the peak systolic and end-diastolic velocities. The type of erectile dysfunction—arterial, venous, or neuronal—was also recorded. Arterial and venous insufficiency were assessed using penile CDUS whereas neurological dysfunction was evaluated using CC-EMG.

### Statistical analysis

Statistical analyses were performed using IBM SPSS Statistics for Windows version 22.0 (IBM Corp., Armonk, New York). Visual (histograms, probability plots) and analytical methods (Shapiro–Wilk's test) were applied to determine the normality of the distributions of the variables. For statistical comparisons of sample percentages between the VG and NVG, the chi-squared and Fisher's exact tests were used. Differences between the groups were assessed using t-tests for independent samples, with p ­values below 0.05 considered statistically significant.

## RESULTS

A total of 97 male patients diagnosed with BD (mean age, 40.4 [10.7] years; disease duration, 12.5 [9.6] years) were included in the study. The groups were comparable in terms of age (p = 0.789), comorbidities (p = 0.718), smoking history (p = 0.847), and the presence of depression (p = 0.254). The VG had a longer disease duration than the NVG (VG: mean [SD], 15.1 [10.9] years vs. NVG: mean [SD], 9.9 [7.3] years; p = 0.007). No significant difference was found between the VG and NVG in terms of BD symptoms, including oral ulcers (p = 1), genital ulcers (p = 0.075), papulopustular lesions (p = 0.229), erythema nodosum (p = 0.079), arthritis (p = 0.240), uveitis (p = 0.612), positive pathergy test (p = 0.536), and HLA-B51 positivity (p = 0.481). Arthralgia was more common in the NVG than in the VG (NVG: n = 36, 75% vs. VG: n = 23, 46.9%; p = 0.005). The groups were similar in terms of tumor necrosis factor alpha inhibitors (p = 0.317), glucocorticoid (p = 0.406), cyclosporine (p = 0.242), and cyclophosphamide (p = 0.204) treatment. The use of azathioprine (p = 0.001), aspirin (p = 0.018), and warfarin (p = 0.012) was significantly higher in the VG than in the NVG. The general characteristics and clinical features of the patients are summarized in Table 1.

**Table 1:** Demographic and clinical characteristics of patients with Behçet's disease

Characteristic		All patients (N = 97)	VG (n = 49)	NVG (n = 48)	p Value
Age, years, mean (SD)		40.4 (10.7)	40.7 (10)	40.1 (11.5)	0.789
Disease duration, years, mean (SD)		12.58 (9.63)	15.14 (10.93)	9.96 (7.33)	**0.007** *
Patients with ≥1 comorbidities, n (%)		32 (33)	17 (34.7)	15 (31.3)	0.718
Comorbidities, n (%)					
	*Hypertension*	12 (12.4)	5 (10.2)	7 (14.6)	0.513
	*Diabetes mellitus*	6 (6.2)	2 (4.1)	4 (8.3)	0.436^a^
	*Coronary artery disease*	7 (7.2)	4 (8.2)	3 (6.3)	0.512^a^
Depression, n (%)		55 (56.7)	25 (51)	30 (62.5)	0.254
Smoking history, n (%)		76 (78.4)	38 (77.6)	38 (79.2)	0.847
Behçet's disease features, n (%)					
	*Oral ulcer*	93 (95.9)	47 (95.9)	46 (95.8)	1^a^
	*Genital ulcer*	58 (59.8)	25 (51)	33 (68.8)	0.075
	*Papulopustular lesion*	63 (64.9)	29 (59.2)	34 (70.8)	0.229
	*Erythema nodosum*	36 (37.1)	14 (28.6)	22 (45.8)	0.079
	*Arthritis*	29 (29.9)	12 (24.5)	17 (35.4)	0.240
	*Uveitis*	48 (49.5)	23 (46.9)	25 (52.1)	0.612
	*Pathergy*	47 (48.4)	26 (53.1)	21 (46.7)	0.536
	*Thrombosis*	47 (48.4)	47 (95.9)	0 (0)	**0** a
	*Aneurysm*	7 (7.2)	7 (14.3)	0 (0)	**0.012** a
	*HLA-B51*	17 (42.5)	7 (50)	10 (38.5)	0.481
	*Arthralgia*	59 (60.8)	23 (46.9)	36 (75)	**0.005** a
Active medical treatment for BD, n (%)					
	*Azathioprine*	34 (35.1)	25 (51)	9 (18.8)	**0.001** a
	*Cyclosporine*	2 (2.1)	0 (0)	2 (4.2)	0.242^a^
	*TNF-α inhibitor*	10 (10.3)	7 (14.3)	3 (6.3)	0.317^a^
	*Glucocorticoid*	28 (28.9)	16 (32.7)	12 (25)	0.406
	*Cyclophosphamide*	6 (6.2)	5 (10.2)	1 (2.1)	0.204a
	*ASA*	29 (29.9)	20 (40.8)	9 (18.8)	**0.018** a
	*Warfarin*	7 (7.2)	7 (14.3)	0 (0)	**0.012** a
International Index of Erectile Function-5					
	*IIEF-5 score ≤ 21, n (%)*	49 (50.5)	24 (49)	25 (52.1)	0.76^a^
	*IIEF-5 score, mean (SD)*	17.8 (7.6)	17.4 (7.9)	18.3 (7.3)	0.581*

ASA: Acetylsalicylic acid; IIEF-5: International Index of Erectile Function-5; HLA-B51: human leukocyte antigen-B51; n: Number; NVG: Non-vascular group; SD: Standard deviation; TNF-α: Tumor necrosis factor alpha; VG: Vascular group.

* Independent samples t test.

a Fisher's exact test

Half of all patients with BD were found to have ED (n = 49, 50.5%). There was no significant difference between the groups in terms of the presence of ED (VG: n = 24, 49% vs. NVG: n = 25, 52.1%; p = 0.76) and IIEF-5 scores (VG: mean [SD], 17.4 [7.9] vs. NVG: mean [SD], 18.3 [7.3]; p = 0.581) (Table 1).

No differences in the presence of oral ulcers (p = 1), papulopustular lesions (p = 0.076), uveitis (p = 0.477), genital ulcers (p = 0.901), arthralgia (p = 0.361), and arthritis (p = 0.876) were observed between patients with BD with and without ED (Table 2). Although no difference in smoking history was observed between patients with BD with and without ED (p = 0.764), depression was more common in patients with ED (n = 33, 67.3%) than in patients without ED (n = 22, 45.8%; p = 0.033).

**Table 2:** Comparison of the presence of arthralgia, arthritis, oral ulcers, papulopustular lesions, uveitis, and genital ulcers based on erectile dysfunction status

Presence of condition	IIEF-5 score ≤ 21 (n = 49)	IIEF score > 21 (n = 48)	p Value
Oral aphtha ever, n (%)	47 (95.9)	46 (95.8)	1^a^
Oral aphtha never, n (%)	2 (4.1)	2 (4.2)	
Papulopustular lesion ever, n (%)	36 (73.5)	27 (56.3)	0.076*
Papulopustular lesion never, n (%)	13 (26.5)	21 (43.8)	
Uveitis ever, n (%)	26 (53.1)	22 (45.8)	0.477*
Uveitis never, n (%)	23 (46.9)	26 (54.2)	
Genital ulcer ever, n (%)	29 (59.2)	29 (60.4)	0.901^a^
Genital ulcer never, n (%)	20 (40.8)	19 (39.6)	
Arthralgia ever, n (%)	32 (65.3)	27 (56.3)	0.361*
Arthralgia never, n (%)	17 (34.7)	21 (43.8)	
Arthritis ever, n (%)	15 (30.6)	14 (29.2)	0.876*
Arthritis never, n (%)	34 (69.4)	34 (70.8)	

IIEF-5: International Index of Erectile Function-5; n: Number. Patients with an IIEF-5 score of 21 or below were classified as having erectile dysfunction.

* Chi-squared test.

a Fisher's exact test.

**Figure 2:**
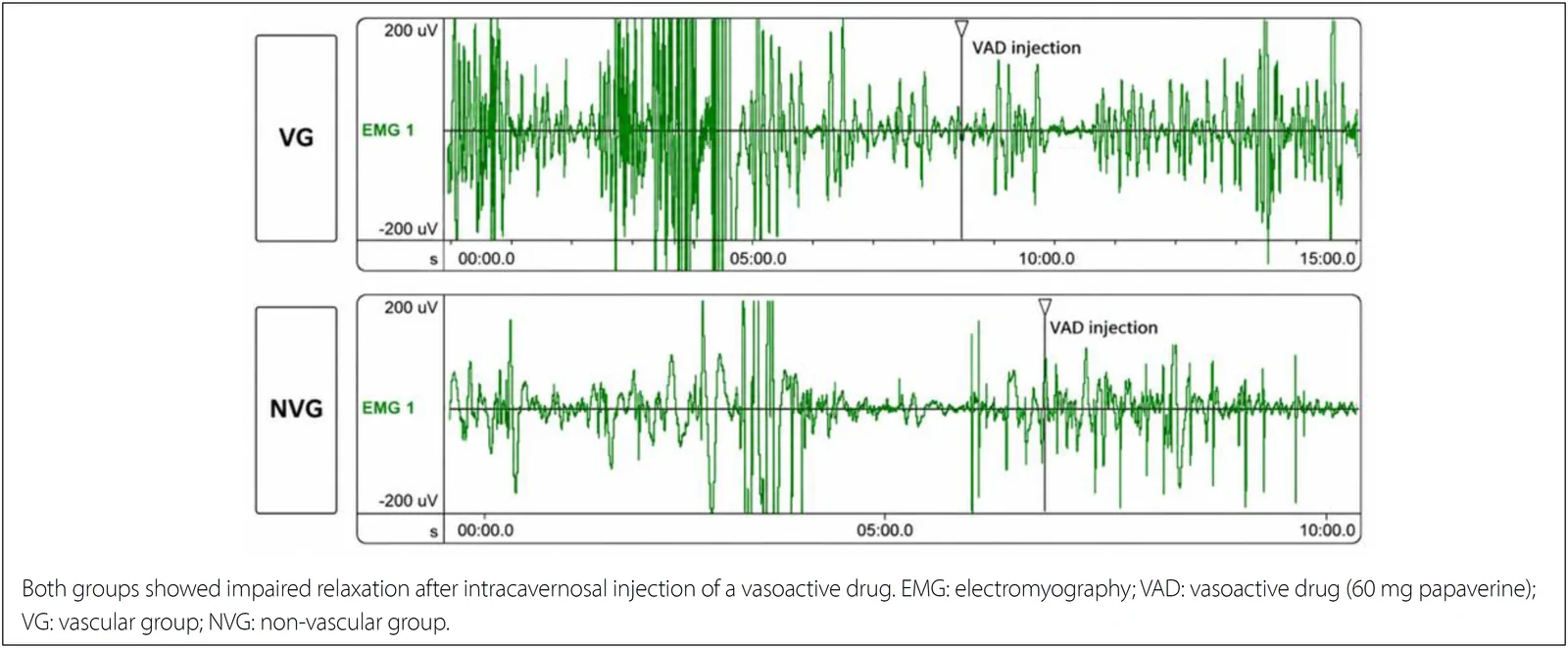
Representative corpus cavernosum electromyography findings in the vascular and non-vascular groups of patients with Behçet's disease.

Of the 24 patients who underwent penile CDUS and CC-EMG, 22 (91.6%) were found to have ED due to arterial or venous insufficiency or neurological problems. The proportion of patients with peak systolic velocities of less than 30 cm/s was significantly higher in the VG than in the NVG (VG: n = 12, 80% vs. NVG: n = 2, 22.2%; p = 0.01). No significant differences were found in the proportions of end-diastolic velocities of more than 3 cm/s (p = 1), peak systolic (p = 0.063), end-diastolic velocities (p = 0.437), and resistive index (p = 0.178) between the groups (Table 3). There was no significant difference in CC-EMG results between the groups (p > 0.05) (Table 3 and Figure 2).

**Table 3:** Results of penile electromyography and color Doppler ultrasonography

Measurement		Total (n = 24)	VG (n = 15)	NVG (n = 9)	p Value
CDUS						
	*Peak systolic,* *n (%)*	14 (58.3)	12 (80)	2 (22.2)	**0.01** a
	*Peak systolic velocity, cm/s, mean (SD)*	34.2 (18.1)	31.66 (19.61)	38.66 (15.53)	0.063*
	*End-diastolic, n (%)*	16 (66.7)	10 (66.7)	6 (66.7)	1^a^
	*End-diastolic velocity, cm/s, mean (SD)*	4.25 (3.3)	4.66 (3.59)	3.55 (2.78)	0.437*
	*Resistive index, mean (SD)*	0.82 (0.116)	0.8 (0.137)	0.86 (0.053)	0.178*
CC-EMG						
	*Pre-VAD amplitude, µV, median (IQR*)	355 (175)	335 (172.5)	390 (210)	0.259^b^
	*Pre-VAD frequency, mean (SD)*	6 (1.8)	5.5 (1.7)	6.8 (1.7)	0.089^b^
	*Post-VAD amplitude, µV, median (IQR)*	215 (200)	230 (220)	160 (150)	0.245^b^
	*Post-VAD frequency, median (IQR)*	5 (2)	5 (2)	6 (1.75)	0.073^b^
	*Relaxation degree, n (%)*	12 (50)	8 (53.3)	4 (44.4)	0.673^a^
	*Relaxation degree, cm/s, mean (SD)*	0.46 (0.25)	0.41 (0.24)	0.54 (0.26)	0.234*
Type of erectile dysfunction, n (%)						
	*Arterial*	14 (58.3)	12 (80)	2 (22.2)	**0.010** a
	*Venous*	17 (70.8)	10 (66.7)	7 (77.8)	0.669^a^
	*Neuronal*	12 (50)	8 (53.3)	4 (44.4)	1^a^

CC-EMG: Corpus cavernosum electromyography; CDUS: Color Doppler ultrasonography; ED: Erectile dysfunction; IQR: Interquartile range; n: Number; NVG: Non-vascular group; VAD: Vasoactive drug; VG: Vascular group. Peak systolic: The proportion of peak systolic velocities of less than 30 cm/s. End-diastolic: The proportion of end-diastolic velocities of more than 3 cm/s.

* Independent samples t test.

a Fisher's exact test.

b Mann–Whitney U test.

## DISCUSSION

In the present study, we found that approximately half of all patients with BD had ED, and that the frequency of ED was similar in patients with vascular and non-vascular involvement. Arterial insufficiency was more common in patients with vascular involvement than in those without vascular involvement, whereas CC-EMG findings were similar regardless of vascular involvement. Furthermore, depression was found to be more common in patients with ED. To the best of our knowledge, this is the first study to investigate the effect of vascular involvement on ED in patients with BD using invasive methods.

Penile erection is a complex process involving neuronal, vascular, hormonal, and cavernous structures.^28^ The parasympathetic center, located in the sacral regions S2 to S4, is activated by sexual stimuli or reflex triggers from touch sensations in the penis through the central nervous system. This center sends signals through preganglionic nerves to the pelvic ganglion, which then sends signals through erectogenic nerves to the penis. These nerves regulate penile blood flow and maintain an erection by releasing mediators.^28^ Damage to any of the components involved in penile erection can disrupt the erection process and lead to ED. Chronic diseases such as hypertension, coronary artery disease, diabetes, hyperlipidemia, malignancy, advanced age, smoking, alcohol consumption, obesity, hypogonadism, hyperprolactinemia, and conditions that damage the mechanism of erection, such as Peyronie's disease and a history of pelvic surgery or radiotherapy, are common causes of ED.^15,29^ Moreover, ED can occur for various reasons in rheumatological diseases, including rheumatoid arthritis, ankylosing spondylitis, Sjögren's disease, and systemic sclerosis. Previous studies have reported that the incidence of ED in BD ranges between 36.8% and 90%.^12,13,30,31^

In patients with BD, the overall mean prevalence of ED ranges from 14% to 48%, with higher rates observed in the United States and in Southeast Asia than in Europe. The prevalence of ED in Germany is 19.2%, while it is 30% in the general European population.^32^ A study conducted in Türkiye found that the prevalence of ED was 49.9% among men aged 40 to 49, with higher rates associated with increasing age, lower education levels, unemployment, diabetes, hypertension, and depression.^33^ The ED rate observed in the present study was 50.5% in patients with BD, with a mean patient age of 40.4 years, consistent with the findings of Akkus et al.^33^

In our cohort, the rate of ED was low in smokers with BD. However, Li et al.^34^ conducted a prospective study in the United Kingdom and determined that smoking has a negative impact on ED. This difference may be related to the patients’ level of exposure to smoking. Li et al.^34^ also suggested that comorbidities such as hypertension, hyperlipidemia, and diabetes have negative effects on ED. In our cohort, there was no significant difference in the prevalence of these comorbidities between patients with ED and those without ED (38.8% vs. 61.2%; p = 0.221). Moreover, patients with and without vascular involvement had similar comorbidity findings. A significant difference in disease duration was observed between the two groups: The longer disease duration in the VG suggests that prolonged disease duration may represent a predisposing factor for the development of endothelial dysfunction of the arteries and vascular damage. Therefore, this difference should be considered a potential confounding factor when interpreting the study findings.

Consistent with the literature,^12,35,36^ we found a higher frequency of ED in patients with depression. Hiz et al.^12^ reported a negative correlation between depression scores and IIEF scores. Similarly, Oliveira et al.^35^ found that depression negatively affects sexual function. The findings of another study suggest that depression has a negative effect on sexual function in premenopausal women with mucocutaneous involvement.^36^ ED and sexual function should be carefully monitored in patients with BD with symptoms of depression. Given the well-established association between depression and ED, depression may have acted as a potential confounding factor in our study. Although performing a multivariable analysis would have been valuable to determine the independent contribution of vascular involvement after adjustment for depression, the present study was not designed for such an analysis. Future studies with larger sample sizes should be conducted to address this issue using multivariable models.

One study showed that medication use was not associated with sexual dysfunction.^12^ Yıldız et al.^37^ found no association between glucocorticoid use and sexual dysfunction. Koçak et al.^38^ obtained similar results in their study involving premenopausal patients with BD. Another study showed that tofacitinib monotherapy was able to reduce the severity of ED and disease activity, and improve health-related quality of life in male patients with Rheumatoid arthritis who presented with ED.^39^ Although our study had no longitudinal data regarding whether erectile function is restored with BD treatment in patients with ED, improvement in ED and quality of sexual life has been reported after treatment with anti-tumor necrosis factor alpha drugs in another chronic systemic inflammatory disease, ankylosing spondylitis. Therefore, erectile function may improve in patients with ED after treatment for BD.^40^ Further studies are needed to investigate the effects on ED of treatment in patients with BD.

In the present study, no relationship was found between the presence of ED and the clinical symptoms of BD, such as oral aphthae, genital ulcers, papulopustular lesions, uveitis, arthralgia, and arthritis; this finding is consistent with those of previous studies.^12,32,37,38^ In one study, oral ulcers, genital ulcers, active skin lesions, and uveitis were not shown to be associated with sexual dysfunction.^12^ Koçak et al.^38^ reported no association between genital ulcers and sexual dysfunction in BD. Similarly, Yıldız et al.^37^ reported no significant relationship between active oral ulcers, genital ulcers, arthritis, and sexual dysfunction. Contrary to these findings, Saur et al.^32^ found that genital ulcers were more common in patients with BD with sexual dysfunction.

The findings of the present study showed that the proportion of patients with arterial insufficiency, as determined by reduced peak systolic velocity measured using penile Doppler ultrasonography, was higher in patients with vascular involvement than in those without. Erdem et al.^30^ classified patients with BD into the mucocutaneous and systemic groups to evaluate sexual dysfunction. The findings of their study showed no significant difference between the two groups in terms of ED scores or penile Doppler ultrasonography results. An increased incidence of ED has been reported in patients with BD who have neurological involvement.^31^ By contrast, Aksu et al.^16^ identified ED in two patients without neurological involvement, with the authors commenting that ED may occur without neurological involvement. In the present study, arterial insufficiency was more common in patients with ED with vascular involvement. Vascular reinvolvement may thus be a cause of ED in patients with BD. The findings of our study provide new insight into vascular involvement and arterial insufficiency as a possible cause of ED.

The IIEF-5 has been shown to be an excellent diagnostic tool, and a cutoff of 21 points yields an 88% specificity and 98% sensitivity.^23^ In a study evaluating tofacitinib monotherapy and ED in rheumatoid arthritis, 17 patients (35.4%) had severe ED, 10 (20.8%) had moderate ED, 10 (20.8%) had mild to moderate ED, and 11 (22.9%) had mild ED, as assessed using the IIEF-5.^39^ Furthermore, a study conducted by the EULAR Scleroderma Trials and Research Group evaluated ED in systemic sclerosis and found that only 23 of 130 men (17.7%) had a normal IIEF-5 score.^41^ Although ED is common among patients with BD, the IIEF-5, a noninvasive assessment tool, identified ED with an overall diagnostic accuracy of 91.6% in our cohort. The presence of ED was confirmed using an invasive method in our study; hence, the IIEF-5 can be used effectively to detect ED in patients with ED.

CC-EMG is a minimally invasive examination that enables the autonomic function of the penis and function of the cavernosal smooth muscle to be evaluated.^42^ In a study in which the etiology of ED in young patients was evaluated using CC-EMG, the authors reported that autonomic dysfunction can cause discoordination patterns and a disrupted relaxation response in CC-EMG.^43^ In another study involving patients who developed ED after contracting coronavirus disease 2019, the authors reported that these patients had abnormal CC-EMG patterns and impaired cavernosal smooth muscle relaxation.^44^ By contrast, in another study in which the variations of CC-EMG patterns in younger and older age groups was evaluated, the authors reported that no significant difference was observed between groups in terms of CC-EMG parameters.^25^ In our study, we found no difference in CC-EMG patterns, including frequency, amplitude, and degree of relaxation, between the vascular and non-vascular BD groups. The literature on CC-EMG studies in patients with BD is limited; therefore, the present study provides insight for future research.

This study had some limitations. First, the longer duration of disease observed in the VG is a limitation. Second, the effect of BD treatment on ED was not investigated in this study due to the study design. No longitudinal data are available regarding whether erectile function is restored with treatment for BD, particularly in patients with arterial ED due to vascular involvement. Third, the sample size of the study was relatively small because not all patients diagnosed with ED through the IIEF-5 score were willing to participate in the CC-EMG and CDUS examinations. However, there are limited studies on sexual dysfunction in the BD population, and the number of participants in these studies is also limited. Therefore, our study has the potential to contribute to literature.

## CONCLUSION

ED is common in patients with BD, and arterial ED is more prevalent among those with vascular involvement. Sexual dysfunction, which is frequently not screened for in patients with BD, should be thoroughly evaluated, and early intervention is recommended. Considering ED in the management of patients with BD may be beneficial for improving their quality of life.

## Data Availability

Data supporting the findings of this study are available upon request from the corresponding author, Hakan Apaydin.
